# Polymer Blend Spiking Method for Quantifying Polypropylene Variants in 100% Polypropylene Blends

**DOI:** 10.3390/polym17182543

**Published:** 2025-09-20

**Authors:** Meysam Hashemnejad, Ami Doshi

**Affiliations:** Cincinnati Technology Center, LyondellBasell, 11530 Northlake Drive, Cincinnati, OH 45249, USA

**Keywords:** polyolefin characterization, compositional analysis, post-consumer recyclate (PCR), polypropylene, polyethylene, crystallization elution fractionation (CEF)

## Abstract

Understanding the type of polypropylene (PP) in post-consumer recycled (PCR) PP is valuable for optimizing mechanical recycling approaches, especially when blending with virgin polymers for specific applications. However, accurately identifying and quantifying the various types of polypropylene, including isotactic Homo-PP (Homo-PP), Random-PP, and non-crystalline PP components (such as xylene-soluble atactic PP and amorphous ethylene–propylene copolymers), presents significant challenges when dealing with materials composed entirely of polypropylene. To address this, we propose a solution-based crystallization elution fractionation (CEF) technique to determine the composition of different PP variants in PP blend systems. Our approach involves introducing a controlled amount of linear low-density polyethylene (LLDPE) into the 100% PP sample in solution, enabling the separation of Homo-PP from Random-PP. By applying established calibration curves, we quantitatively resolve the content of Homo-PP, Random-PP, and non-crystalline PP. The calibration is effective across the full composition window, enabling accurate quantification of Random-PP and Homo-PP from pure (100%) components to mixed systems spanning ~5 wt.% to 95 wt.% Random-PP. This comprehensive analysis offers valuable insights into the distribution of PP variants within the material, facilitating informed decision-making in recycling and material selection processes, ultimately enhancing the efficiency and sustainability of PP recycling operations.

## 1. Introduction

As ambitions for plastic recycling have increased across various industries, ranging from plastic production to processing to commodity goods manufacturing, companies are increasingly motivated to address the harmful environmental impact of used plastics inadvertently released into the environment [1,2,3,4,5,6,7]. This includes both mitigating environmental damage and harnessing the valuable resource of post-consumer recycling materials. One major challenge in plastic recycling is effectively sorting various polymer types after collection [8,9,10,11,12]. As industries advance in automated sorting techniques, such as machine learning-guided near-infrared spectroscopy, instances of mixed polymer blends will occur [13]. This can happen due to the original composition of products using different polymers or sorting challenges leading to blends of polymer recyclates. For instance, post-consumer recycled polypropylene (PP) or polyethylene (PE) may contain a mixture of various PP or PE types, or it could include unwanted polymer contamination in post-consumer recyclates [14,15,16]. Post-consumer PP and PE polymers make up a significant portion of recycled plastics, being among the most commonly used polymers due to their favorable mechanical properties and processability [8,9,17,18]. These materials are often melt-blended with virgin polymers at different ratios as part of a mechanical recycling approach, which is currently a major recycling method [11,19]. This trend is propelled by the growing adoption of post-consumer polymers in various commodity products, frequently labeled with a percentage of post-consumer recycled content [8]. However, before PCR polymers are melt-blended with virgin polymer, thorough analytical analysis is required to assess the polymer composition and the presence of polymeric/non-polymeric contaminants, considering that proper separation, washing, and filtration have been applied throughout PCR polymer fabrication [10,11,15,16,19,20]. For example, if the ratio of Random-PP to Homo-PP in the post-consumer recycled PP feedstock is not properly controlled or understood, the resulting recycled material may lack the desired properties for specific applications.

Polypropylene has different variants, namely Homo-PP, Random-PP, and high-impact PP [21,22,23,24]. Polypropylene homopolymers are predominantly produced through the Ziegler–Natta polymerization process, yielding isotactic Homo-polypropylene (Homo-PP) with high chain regularity and crystallinity, resulting in high crystallization temperatures [22,25]. Homo-PP types, including isotactic, syndiotactic, and atactic forms, differ in the spatial arrangement of propylene monomers [22]. Isotactic PP (i-PP) is the most common, used in rigid packaging, consumer goods, textiles, automotive components, and medical devices. Syndiotactic PP, synthesized using metallocene, has a distinct spatial arrangement, while atactic PP is mostly amorphous. Random polypropylene (Random-PP), which contains less than 6% ethylene as a comonomer, has a random arrangement of ethylene and propylene monomer units. This disrupts crystallinity, resulting in a lower crystallization temperature compared to Homo-PP. Random-PP is often used in applications that require clarity and flexibility due to its lower crystallinity and reduced melting point compared to isotactic Homo-PP (Homo-PP). This makes it ideal for producing clear, flexible food packaging films that need to maintain a certain level of transparency and flexibility. High-impact polypropylene or heterophasic copolymer polypropylene (HECO-PP) blends ethylene-rich ethylene/propylene copolymers with isotactic Homo-PP or Random-PP, creating an amorphous phase that imparts rubbery behavior and impact resistance. The ethylene/propylene rubber (EPR) content in these blends ranges from 5% to over 70% by weight [26].

Understanding the composition of post-consumer recycled PP is valuable because it may contain various types of PP, including isotactic Homo-PP, Random-PP, and non-crystalline PP (xylene-soluble fraction, a traditional method in the industry for measuring the amount of rubbery phase in semi-crystalline polypropylene). Determining the ratio of Homo-PP to Random-PP in PCR polymers is important for optimizing their incorporation into feedstocks. This understanding enables better control over the properties of the resulting blends of PCR and virgin polymer, ensuring that they meet the required specifications for specific applications. Additionally, understanding the type of PP present in PCR polymers aids in tailoring processing conditions and improving the overall quality and performance of recycled materials. For instance, a higher-than-expected proportion of Homo-PP in a blend intended for flexible films could result in a product that is too rigid, lacks the necessary clarity, and has poor performance in the intended application. Conversely, a higher proportion of Random-PP in a blend intended for rigid applications could lead to a product that is too flexible and lacks the necessary structural integrity.

Several compositional analysis methods have been reported to characterize and quantify polypropylene content (primarily focusing on isotactic polypropylene, i-PP) in polyethylene (PE) blends or vice versa [23,24,27,28,29,30,31,32,33,34,35,36]. However, these methods do not address the quantification of different PP types within 100% PP systems. Recently, we introduced a solution-based crystallization elution fractionation (CEF) methodology capable of quantifying isotactic Homo-PP, Random-PP, and non-crystalline PP (i.e., xylene-soluble fraction) in HDPE blends through a single experiment [26]. Building on this approach, we have extended the CEF technique to enable quantification of different PP types in 100% PP materials. This adapted technique enables precise determination of PP variants in PP blend systems. By spiking the 100% PP materials with linear low-density polyethylene (LLDPE), we make the separation of Homo-PP and Random-PP possible during CEF. In the absence of LLDPE, these components elute simultaneously with significant peak overlap. The modified elution behavior introduced by LLDPE enables quantification of Homo-PP and Random-PP using established calibration curves, while non-crystalline PP is inherently detected as the soluble fraction in the CEF profile.

## 2. Experiments

### 2.1. Materials

The polymer samples used in this study were sourced from commercially available grades supplied by LyondellBasell Industries N.V. (see Table 1 for details). The table outlines key attributes including weight-average molecular weight (Mw) and molecular weight distribution (MWD), both measured via GPC/SEC. The proportion of non-crystalline material was estimated from the soluble fraction identified through CEF analysis. Methyl group concentration (CH_3_ per 1000 total carbon atoms) was determined using CEF, and melt flow rate (MFR), also referred to as melt index, was evaluated under standard testing conditions.

The Random-PP materials used in this work typically contain ~3.5 wt.% ethylene, based on reactor target values. Ethylene content is generally assessed using FTIR, with calibration derived from NMR data. For CEF testing, both binary and tertiary blends were prepared by weighing and mixing the appropriate quantities of LLDPE and various PP grades in 1,2,4-trichlorobenzene (TCB) solvent.

### 2.2. Crystallization Elution Fractionation (CEF)

To analyze the chemical composition distribution of polymers, a CEF instrument (Polymer Char, Valencia, Spain) was utilized [37]. Approximately 25 mg of sample was weighed into a 10 mL vial and sealed for automated processing. Dissolution was carried out using an Agilent autosampler in 8 mL of 1,2,4-trichlorobenzene (TCB) containing 300 ppm butylated hydroxytoluene as an antioxidant, maintained at 160 °C for one hour.

A 400 µL aliquot of the dissolved sample was then injected into the CEF column, pre-equilibrated at 105 °C. During the crystallization stage, the column was cooled from 105 °C to 30 °C at a rate of 2 °C/min under a reduced TCB flow (~42 µL/min) to promote fractionation and minimize co-crystallization. In the subsequent elution stage, the temperature was increased from 30 °C to 160 °C at 4 °C/min while raising the TCB flow rate to 1 mL/min.

The eluted fractions were analyzed at 160 °C using an IR5 detector to monitor concentration and composition. Data analysis was performed using Polymer Char’s CEF One software, and peak areas in the elution profiles were quantified using OriginPro 8.6.

Soluble fraction values were determined using the CEF technique. Melt index measurements were conducted at 190 °C/2.16 kg for PE and 230 °C/2.16 kg for PP. Weight-average molecular weight (Mw) and molecular weight distribution (MWD) were measured using high-temperature GPC or SEC. The methyl group content (CH_3_/1000TC) was quantified through online infrared detection integrated with the CEF system.

### 2.3. Differential Scanning Calorimetry (DSC)

Thermal behavior of the polyolefin blends was examined using a DSC Q1000 instrument (TA Instruments, New Castle, DE, USA). Each sample underwent a standard thermal cycle beginning with heating from ambient temperature to 160 °C for polyethylene or 220 °C for polypropylene and PP/PE blends, aimed to eliminate prior thermal history. This was followed by cooling to 0 °C at a rate of 10 °C/min to capture the crystallization temperature (T_c_). A second heating phase at the same rate was used to determine the melting temperature (T_m_).

Blends of PE and PP were prepared at varying concentrations using a Thermo Scientific HAAKE MiniLab 3 micro-compounder operated at 190 °C. To confirm blend composition, CEF analysis was performed, showing consistent results between melt-compounded samples and those assembled by manually weighing and placing the individual components directly into CEF vials.

## 3. Results and Discussion

**Figure 1:**
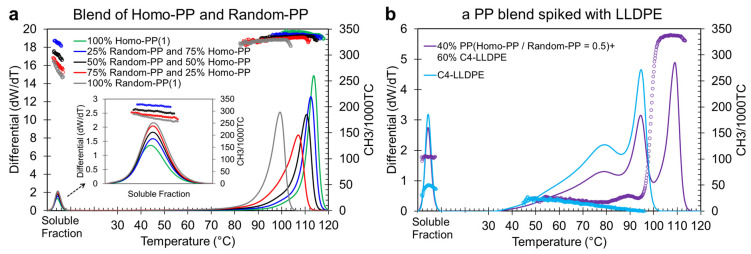
CEF profiles of polypropylene (PP) blend systems. (**a**) Differential function curves and CH_3_/1000TC values plotted against elution temperature for Homo-PP, Random-PP, and blends containing 25%, 50%, and 75% Homo-PP. The inset highlights the soluble fraction, representing non-crystallized polymer components. (**b**) Elution profile and CH_3_/1000TC data for a 50/50 Homo-PP/Random-PP blend spiked with C4-LLDPE to achieve a final composition of 60% LLDPE and 40% PP blend by weight. Pure LLDPE data is included for comparison. The lines represent the elution profiles, while the symbols indicate CH_3_/1000TC values, with both elements color-coded consistently for each polymer system.

Determining the relative amounts of Random-PP and Homo-PP within blend systems presents significant challenges due to the similarity in their molecular structures. Specifically, these semi-crystalline PPs co-crystallize when present together in a blend, making it nearly impossible to differentiate between the two. Figure 1a displays the CEF elution profiles and their corresponding CH_3_/1000TC signals for Homo-PP and Random-PP blends with varying concentrations, ranging from pure Homo-PP to 25%, 50%, and 75% Random-PP, and finally to pure 100% Random-PP. As illustrated, the elution profiles of Random-PP and Homo-PP blends elute as a single peak above room temperature, positioned according to their composition between that of pure Random-PP (lower elution temperature at ~99 °C) and pure Homo-PP (higher elution peak temperature at 114 °C). Their CH_3_/1000TC values are also very close, ~320 for Random-PP and 339 for Homo-PP, which does not aid in differentiating between these PPs.

Differentiating Random-PP from Homo-PP using conventional differential scanning calorimetry (DSC) is similarly challenging due to their closely related crystallization and melting behaviors. The structural similarity between these two semi-crystalline polypropylene types leads to co-crystallization when blended, resulting in overlapping thermal transitions. As shown in the DSC thermograms provided in the Supporting Information (Figure S1), blends of Homo-PP and Random-PP exhibit broad, single crystallization and melting peaks that shift gradually with blend composition, without any distinct thermal signatures that would enable deconvolution of the individual components. For instance, the melting endotherms range from ~161 °C for pure Homo-PP to ~146 °C for pure Random-PP, yet no separate peaks appear at intermediate compositions, as expected. This thermal overlap underscores the limitations of DSC in quantifying or even qualitatively distinguishing between Homo-PP and Random-PP in blend systems.

Moreover, the presence of amorphous PE-PP compounds further complicates compositional analysis by DSC. While DSC is a versatile technique that is widely accessible and easily implemented at manufacturing sites, it has a major limitation for blend analysis: it provides little to no practical insight into the amorphous fraction. In particular, if the PP blend contains a significant amount of amorphous PE-PP material, this phase remains undetected, and its contribution to the overall composition cannot be quantified. Although the change in heat capacity (ΔCp) at the glass transition temperature (Tg) can, in principle, be correlated with the amount of amorphous content [24], this approach requires accurate reference values for ΔCp of fully amorphous PE-PP or atactic PP. Factors such as molecular weight, comonomer content and type, and tacticity can significantly affect the measured ΔCp, making it difficult to apply a universal reference. These constraints significantly limit the utility of DSC for comprehensive compositional analysis in complex polyolefin systems.

Quantifying different polypropylene (PP) variants, particularly Homo-PP and Random-PP, in 100% PP systems presents a significant analytical challenge. These components tend to co-crystallize and elute simultaneously in solution-based polymer fractionation techniques such as CEF, producing a single, indistinguishable peak. In essence, the problem lies in the sample itself, its compositional complexity renders it analytically non-resolvable. Unlike DSC, where the melting and crystallization peaks of Homo-PP and Random-PP strongly overlap and prevent quantitative resolution, the CEF approach leverages both elution separation and chemical specificity. By introducing LLDPE as a spike, Random-PP can be distinguished from Homo-PP through branching-sensitive CH_3_/1000TC information, thus overcoming the fundamental limitation of DSC. To implement this, we drew inspiration from analogous spiking strategies in other fields, specifically, the well-established technique of sample spiking or standard addition [38]. In bioanalytical method development, spiking with known analyte concentrations ensures that added compounds undergo the same extraction and detection processes as native analytes, enabling accurate calibration in complex matrices, demonstrated in the validation of LC–MS/MS methods for DHA quantification in blood serum [39]. Similarly, in environmental soil science, Doick et al. investigated how different spiking approaches for phenanthrene in field-moist soils influenced the formation of non-extractable residues [40]. Their work underscores the importance of carefully designed spiking techniques to achieve reproducibility and homogeneity in heterogeneous matrices [40].

Inspired by these precedents, we developed a novel CEF–spiking strategy to address the challenge of resolving Homo-PP and Random-PP in 100% PP blends. By introducing a known quantity of linear low-density polyethylene (LLDPE) into the PP sample in solution, we intentionally modify the crystallization behavior during CEF analysis. This alteration enables the separation of overlapping Homo-PP and Random-PP peaks, which are otherwise indistinguishable in standard CEF profiles. In this approach, spiking transforms a previously intractable system into one that can be effectively resolved and quantified using conventional CEF.

In our protocol, we use C_4_-LLDPE (a C_2_–C_4_ random copolymer) as the spiking agent at a fixed concentration of 60 wt.%, with the remaining 40 wt.% consisting of the PP blend under investigation. This 60% LLDPE level was found to be optimal for effectively distinguishing between Homo-PP and Random-PP across various blend ratios, while maintaining sufficient sensitivity for quantifying the PP components.

To illustrate this, Figure 1a shows the PP blends before spiking, while Figure 1b presents the differential CEF profile of a representative 50/50 Homo-PP and Random-PP blend spiked with C_4_-LLDPE. The elution profile of pure Ziegler–Natta LLDPE is also included as a reference. Pure ZN-LLDPE typically exhibits a broad, low-temperature peak around 80 °C, corresponding to highly branched polymer chains, followed by a second peak at ~95 °C, indicating more linear, thicker lamellae. The CH_3_/1000TC signal decreases linearly from ~22 at 50 °C to near zero at ~95 °C, reflecting decreasing branch content. In the spiked PP blend (50/50 Homo-PP/Random-PP), the elution curve resembles that of LLDPE but reveals a third distinct peak at ~110 °C, attributed to Homo-PP. The CH_3_/1000TC signal also provides compositional insight. From 50 °C to 70 °C, it overlaps with pure LLDPE, then begins to diverge due to Random-PP, which shares a similar elution range but differs in branching (CH_3_/1000TC). A sharp increase between 95 °C and 105 °C, reaching a saturation value near 333, marks the presence of Homo-PP. This analysis confirms that spiking facilitates resolution. Homo-PP emerges as a high-temperature peak, while Random-PP is partially resolved from LLDPE via its branching signature.

**Figure 2:**
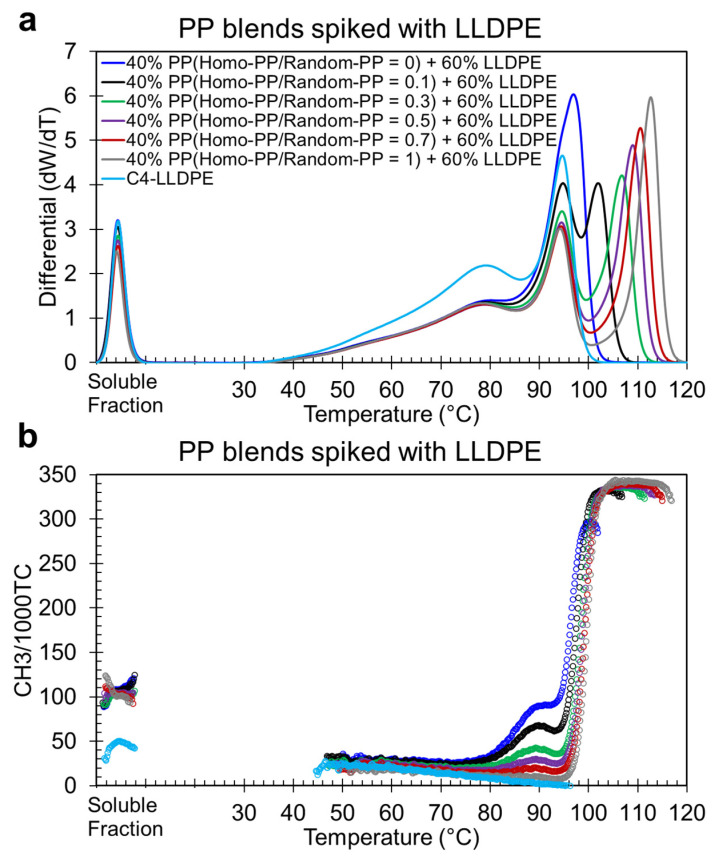
CEF elution profiles of Homo-PP(1) and Random-PP(1) blends spiked with C4-LLDPE. (**a**) Differential function and (**b**) corresponding CH_3_/1000TC signals for a blend of 40 wt.% PP and 60% LLDPE. The 40 wt.% PP component consists of varying concentrations of Homo-PP, ranging from 0 wt.% to 5%, 10%, 20%, and so on, up to 90% and 95 wt.%, as well as pure Homo-PP. Only selected blends are presented for demonstration purposes, with other concentration blends omitted to reduce clutter. All concentrations are by weight. The lines represent the elution profiles, while the symbols indicate CH_3_/1000TC values, with both elements color-coded consistently for each polymer system.

To extend this method to quantification, we applied the spiking technique to blends of varying Homo-PP/Random-PP ratios, ranging from 0% Homo-PP (pure Random-PP) to 100% Homo-PP, including intermediate concentrations of 5%, 10%, 20%, and 95%. In all samples, the PP blend was maintained at 40 wt.%, with LLDPE fixed at 60 wt.%. The resulting differential CEF profiles (Figure 2a) and CH_3_/1000TC signals (Figure 2b) form the basis for calibration.

Several key observations emerge. The LLDPE peak (~79 °C) remains consistent across all samples, confirming that varying PP composition does not affect its elution behavior. The Homo-PP peak shifts progressively lower as Random-PP content increases, decreasing from ~113 °C (pure Homo-PP) to ~102 °C at a Homo-PP/Random-PP ratio of 0.1. All tertiary blends (PP in LLDPE) exhibit a third elution peak above 100 °C, which is absent when only Random-PP is present (i.e., when Homo-PP/Random-PP = 0).

Although spiking introduces sufficient contrast to distinguish Homo-PP as a separate peak, baseline separation is not fully achieved. As the Random-PP content increases, its overlap with Homo-PP becomes more pronounced, as reflected by the elevated valley (at ~98–100 °C) between their elution regions. In all tertiary blend systems, the valley between the Random-PP and Homo-PP elution regions remains above zero, and its magnitude increases with the proportion of Random-PP. For example, in a blend with a 0.10 Homo-PP/Random-PP ratio, the valley between the second and third peaks is significantly elevated, indicating partial coelution of Random-PP as Homo-PP begins to elute above 100 °C. These non-zero valleys reflect the coelution behavior, where some Random-PP elutes alongside Homo-PP. Recognizing this overlapping is important and is accounted for in our calibration strategy, as discussed in the following section.

**Figure 3:**
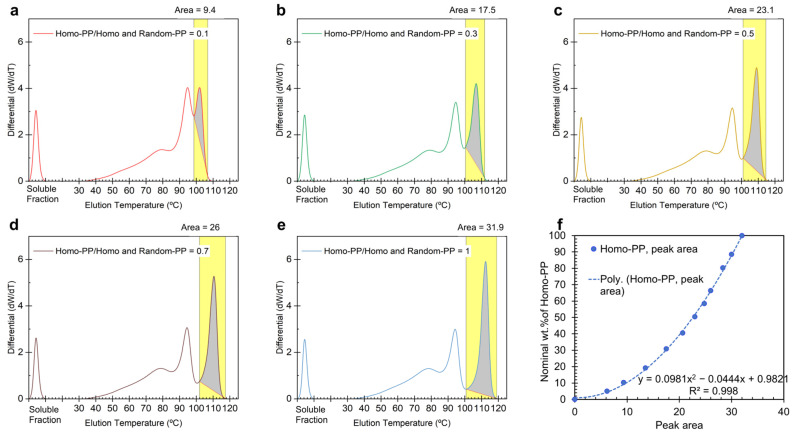
CEF profile of a tertiary blend system. Panels (**a**–**e**) show the differential function vs. temperature for Homo-PP and Random-PP spiked into LLDPE at a fixed concentration of 60 wt.%. Panel (**f**) presents the CEF calibration curve, where the nominal Homo-PP content in the blends is plotted against the calculated area under the Homo-PP elution peak (third peak above room temperature). The ratios in (**a**–**e**) and the nominal wt.% values in (**f**) represent those of the original pure PP prior to spiking into LLDPE. The dotted line in panel (**f**) represents a 2nd order polynomial fit to the experimental data. Yellow-highlighted segments indicate the temperature range used for integration.

To calibrate the wt.% of Homo-PP in PP blends, we measured the area under the third elution peak after spiking with LLDPE (Figure 1b and Figure 2a). Since the peaks are not baseline-separated, we defined the baseline as the line connecting the minima between the 2nd and 3rd elution peaks (where CH3/1000TC is above 300) and the end of the elution signals at the highest temperature. The results for selected concentrations are shown in Figure 3, with the full data set available in the Supplementary Information. The area for Homo-PP is shaded in gray and the integration range is highlighted in yellow (Figure 3a–e).

For calibration, the entire CEF profile, including the soluble fraction, was considered to determine the wt.% of Homo-PP. The nominal Homo-PP content from the 100% PP blends was then correlated with the calculated weight percent derived from the highest-temperature elution peak (above 98 °C), generating the CEF calibration curve (Figure 3f). Interestingly, the data fit best with a second-order polynomial. The (0,0) point indicates that if no third elution peak appears above room temperature when PP blends are spiked with LLDPE, the Homo-PP concentration is zero. Although a linear fit from (0%,0%) to the maximum value at ~32% area for 100 wt.% Homo-PP might be anticipated, the calibration yielded higher values. This outcome suggests that the calibration in Figure 3f may incorporate overlapping components, i.e., Random-PP, in the calculated area. This nuanced calibration approach is strategically advantageous for quantifying Homo-PP content in a 100 wt.% PP blend, ensuring its applicability across various blend systems with different PP components.

To calibrate the content of Random-PP in 100 wt.% PP blends, the CH_3_/1000TC signal was integrated into the area calculation under the elution profile, as Random-PP co-elutes with LLDPE when blended with it. Specifically, in a system where pure Random-PP is spiked into LLDPE (dark blue signal in Figure 2a), the two polymers elute together, but their contributions can be distinguished using CH_3_/1000TC data from the online IR detector (Figure 2b). For pure LLDPE, the CH_3_/1000TC value decreases linearly from ~22 at 50 °C to 0 at around 95 °C. This linear trend is assumed to remain unchanged when PP blends are spiked into LLDPE.

**Figure 4:**
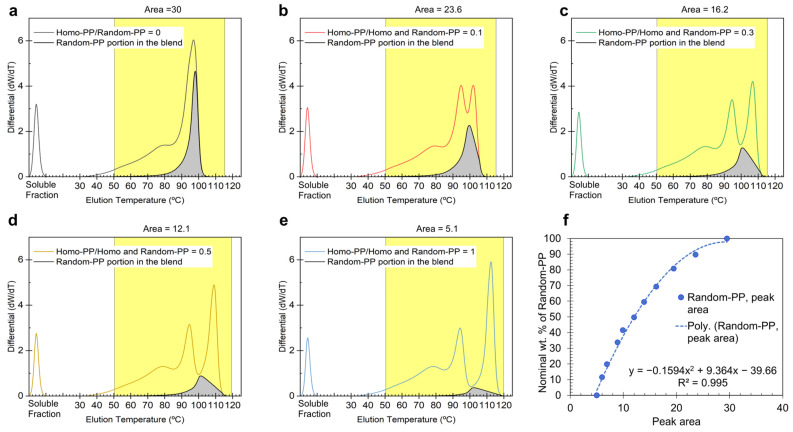
CEF results of tertiary blend systems. CEF profiles of tertiary blend systems. Panels (**a**–**e**) display the differential function vs. temperature for Homo-PP and Random-PP spiked into LLDPE at a fixed concentration of 60 wt.%. Panel (**f**) presents the CEF calibration curves, where the nominal Random-PP content in the PP blends is plotted against the area of the Random-PP elution peak (Random-PP peak area highlighted in gray). The ratios in (**a**–**e**) and the nominal wt.% values in (**f**) correspond to the original pure PP prior to spiking into LLDPE. The dotted line in panel (**f**) represents a 2nd order polynomial fit to the experimental data. Yellow-highlighted segments indicate the temperature range used for integration.

To quantify the Random-PP content within LLDPE matrices (Figure 4a), we employed a differential analysis method incorporating a CH_3_/1000TC normalization factor ranging from 0 to 1. This factor was derived by subtracting the linear baseline of LLDPE from the CH_3_/1000TC value at each elution slice, then normalizing against the maximum CH_3_/1000TC signal observed for pure Random-PP (~320). The weighted area was computed using the following expression:

(1) $$\mathrm{Area}\;=\sum \;A_{s}\times \frac{{\left({\mathrm{CH}}_{3}/1000\mathrm{TC}\right)}_{s}-{\left({\mathrm{CH}}_{3}/1000\mathrm{TC}\;\mathrm{for}\;\mathrm{LLDPE}\right)}_{s}}{320}$$

where *A*_s_ denotes the elution slice area, and the CH_3_/1000TC values are obtained via online IR detection and processed through the CEF software.

In blend systems containing both Homo-PP and Random-PP (Figure 4b–e), a third elution peak emerges at elevated temperatures (>100 °C), indicative of Homo-PP. Due to partial overlap with adjacent Random-PP and LLDPE peaks, direct baseline separation is not feasible. To address this, we defined the integration boundaries by drawing a baseline from the local minimum (~98–100 °C) to the point where the differential signal returns to zero. This approach enabled a more accurate estimation of the Homo-PP peak area despite co-elution effects.

In blends with increasing Random-PP content, however, the baseline initially established for Homo-PP is repurposed as a new differential Y-value, reflecting the assumption that part of the Random-PP co-elutes with Homo-PP. Consequently, when calculating the Random-PP area, the integration range is extended beyond the valley between the second and third peaks and up through the Homo-PP peak. This adjustment accounts for Random-PP co-eluting with Homo-PP, as indicated by the rising baseline with higher Random-PP levels. Importantly, the total Random-PP area comprises two regions: the portion eluting below 100 °C alongside LLDPE, distinguished and quantified using CH_3_/1000TC data, and a fraction that co-elutes with Homo-PP above 100 °C. The combined area of these regions, shown as the gray-shaded area in Figure 4, was used to construct a calibration curve by plotting it against the nominal Random-PP concentration in the blend.

Notably, the calibration curve for Random-PP (Figure 4f) exhibits a concave shape, in contrast to the convex trend observed for Homo-PP (Figure 3f). This opposing curvature suggests a compensatory relationship between the two measurements. The concavity of the Random-PP plot corrects for the systematic overestimation observed in the Homo-PP calibration. An offset is also observed at 0% nominal Random-PP concentration, where the area corresponding to Random-PP remains nonzero (gray region in Figure 4e for the 100% Homo-PP blend). This baseline offset arises from the co-elution of a portion of Homo-PP into the lower-temperature range typically associated with Random-PP, leading to an artificial Random-PP signal even when none is present.

These systematic deviations, i.e., overestimation for Homo-PP and underestimation for Random-PP, are evident from the curvature of the calibration plots. They reflect the physical reality that a fraction of the Random-PP elutes over the same temperature range as Homo-PP, and vice versa. By incorporating these effects into the construction of the calibration curves, the methodology accounts for the co-elution behavior and enables accurate compositional quantification across a range of blend ratios.

**Figure 5:**
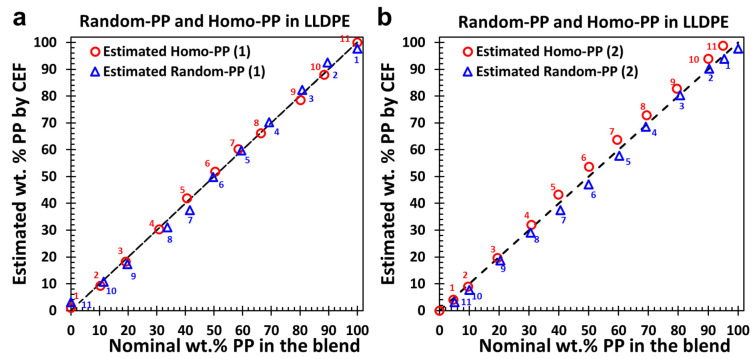
Validation of calibration curves for Homo-PP and Random-PP quantification. (**a**) Estimated vs. nominal wt.% of Homo-PP and Random-PP in the PP blends originally used to construct the calibration curves (Figure 3f and Figure 4f), spiked with 60 wt.% LLDPE. (**b**) Results for a secondary set of blends containing Homo-PP(2) and Random-PP(2) at varying ratios. Reported values are normalized to 100% PP, excluding the LLDPE fraction. Identical symbol labels in (**a**,**b**) denote the same blend systems (e.g., red circle #1 and blue triangle #1 represent the same blend). Symbols in panel (**b**) without numbers indicate 100% Random-PP (triangle) and 0% Homo-PP (circle) within the same blend.

To assess the reliability of the calibration curves (Figure 3f and Figure 4f), we applied the spiking approach to quantify the proportions of different PP types in 100% PP blend systems. As an initial validation step, the calibration curves were tested against the same blends from which they were derived, Homo-PP quantification using Figure 3f and Random-PP quantification using Figure 4f. The outcomes are presented in Figure 5a for blends spiked with a fixed 60 wt.% of LLDPE. In this representation, the PP fractions are normalized relative to the original 100% PP blend prior to spiking. The close alignment between the estimated and nominal values for both PP types demonstrates the reliability of the calibration approach when applied to its source blends, and supporting its extension to other blend systems.

To further validate the calibration curves (Figure 3f and Figure 4f), we applied them to a new blend configuration featuring alternative polypropylene variants. In this case, Homo-PP and Random-PP were replaced with counterparts exhibiting different melt flow rates, referred to as Homo-PP(2) and Random-PP(2). The estimated PP contents, shown in Figure 5b, are compared against their nominal values across blends containing both PP types at varying ratios. Although the blends were spiked with LLDPE to enable differentiation and quantification, the data are normalized to represent a 100% PP basis. The results reveal strong agreement for Homo-PP, with a slight tendency toward overestimation at certain concentrations relative to the 1:1 reference line. In contrast, Random-PP shows a modest underestimation. These findings support the continued applicability of the calibration curves for quantifying PP components in pure PP blend systems.

One of the major advantages of solution-based CEF compositional analysis is its ability to isolate and quantify the non-crystalline portion of polypropylene (PP), which elutes as a soluble fraction. This fraction, often associated with rubbery domains, can be readily separated from the crystalline portion during the elution process. Typically, Homo-PP exhibits a relatively low soluble fraction (around 2–4 wt.%), while Random-PP shows a higher soluble fraction due to the random incorporation of comonomers (i.e., ethylene), which disrupts the regularity of the crystalline structure and lowers crystallinity. The molecular weight distribution also plays a role, as lower molecular weight “waxy” components are more likely to elute in the soluble region.

When a spiking agent like LLDPE is introduced into the solution, its contribution to the total soluble fraction must be accounted for and excluded. For instance, the 60 wt.% LLDPE used in our spiked blends contributes ~6 wt.% to the total soluble fraction, which must be subtracted to isolate the contribution of non-crystalline PP. The remaining soluble content, after subtracting the LLDPE contribution, can be attributed to the non-crystalline PP portion, which includes soluble Homo-PP, Random-PP, or other non-crystalline PP domains present in the blend. Although not the focus of this study, this principle also applies to blends containing heterophasic copolymer PP (HECO-PP), where a significant rubbery phase may exist. In previous work, we demonstrated this capability by introducing HECO-PP into blend systems, where the non-crystalline (soluble) fraction was used to estimate the rubber phase content [26]. The HECO-PP materials, known for their impact strength, include both crystalline Homo-PP (eluting above 110 °C) and non-crystalline PP components that elute in the soluble range. There, we used a CH_3_/1000TC threshold (>150) to help confirm the identity of the non-crystalline PP portion. A similar approach can be employed here: by subtracting the known LLDPE contribution, the remaining soluble material, often more than 2 wt.%, can be confidently assigned as non-crystalline PP.

We selected LLDPE as a more effective spiking material for resolving the co-elution between Random-PP and Homo-PP compared with other polyethylene types such as LDPE and HDPE. Importantly, we consider LLDPE to act primarily as a non-interfering marker rather than as a co-crystallizing component with Random-PP. In typical CEF experiments, Random-PP elutes at slightly lower temperatures than Ziegler–Natta Homo-PP. However, when both are present in a blend, they co-elute as a broad, indistinguishable peak, with the CH_3_/1000TC signal saturated and lacking separation (Figure 1a). By introducing LLDPE into the system, the elution profile gains a distinct reference signal: Random-PP, which partially overlaps with LLDPE, remains distinguishable through CH_3_/1000TC information (used successfully for calibration), while Homo-PP appears as a separate high-temperature peak. Thus, LLDPE provides a reliable baseline against which PP variants can be resolved. In contrast, LDPE is unsuitable as a spiking agent because it elutes at much lower temperatures (typically ~75 °C), far removed from the PP fractions, and therefore does not aid in decoupling Random-PP from Homo-PP. HDPE, while eluting at the same peak temperature as Random-PP, produces a sharp and narrow peak. Our results show that LLDPE offers superior performance, particularly at higher Random/Homo-PP ratios, where HDPE spiking led to stronger overlap between the two PP components compared to LLDPE.

One potential limitation of the current calibration is its sensitivity to the comonomer content in Random-PP. The two Random-PP samples used in this study contain ~3.5–4.2 wt.% ethylene, which reflects a relatively common composition range for reactor-grade Random copolymers. For Random-PPs with significantly lower ethylene content, the crystallization behavior shifts closer to that of Homo-PP, potentially resulting in increased co-elution, particularly after dilution with LLDPE, which can further narrow the resolution window between the two components. This overlap may reduce the separation efficiency of the CEF profile and compromise the accuracy of quantification. Since the core objective of this approach is to characterize and quantify PP components without requiring prior compositional information, this aspect presents an inherent challenge. Without testing and validating the current calibration against Random-PP samples with significantly lower comonomer levels, we cannot fully assess the method’s performance under those conditions. Therefore, we advise that this uncertainty be treated with caution when analyzing unknowns. To ensure analytical accuracy in such cases, further work is recommended to evaluate the calibration’s robustness across a broader range of Random-PP compositions, particularly those with lower ethylene content. This would support the development of a more universally applicable framework and help define the operational boundaries of the current methodology for real-world use cases.

The CEF-IR spiking approach offers a practical route to separate and quantify different polypropylene components, namely Homo-PP, Random-PP, and the non-crystalline fraction that appears in the soluble portion of a typical CEF run. It should be noted that this soluble fraction may also include contributions from low-molecular-weight or waxy chains in addition to truly amorphous PP domains. By integrating the spiking methodology with calibration curves, the technique enables resolution of overlapping elution features, making it possible to distinguish Random-PP from Homo-PP in blend systems. Although further validation is required for materials with unknown or highly variable comonomer levels, particularly in Random-PP, the strategy demonstrates promise as a targeted analytical tool for structured studies and quality control. While HECO-PP and other rubber-modified grades were not the main focus here, their composition (a mixture of amorphous/rubbery domains, Homo-PP, and/or Random-PP) typically gives rise to both an increased soluble fraction and crystalline fractions that coincide with regions covered by the calibration framework. This highlights the broader applicability of the CEF methodology for more complex PP systems.

## 4. Concluding Remarks

This work establishes a practical framework for the compositional analysis of complex polypropylene blends using a solution-based CEF-IR method enhanced by a controlled spiking strategy. By introducing a known quantity of LLDPE into the PP blend in solution, the method transforms a previously unsolvable co-elution problem, where Random-PP and Homo-PP overlap, into a system that can be quantitatively calibrated. The combination of methyl group content (CH_3_/1000C) and elution behavior provides complementary and guiding information in the CEF run, allowing for effective differentiation between PP types based on their elusion profiles. Moreover, the intrinsic measurement of the soluble fraction in CEF offers direct insight into the presence of non-crystalline PP domains, such as amorphous atactic PP or rubbery ethylene–propylene segments. This approach is expected to be particularly valuable for characterizing PCR-PP blends, where quantifying the relative proportions of different PP types is valuable for sorting, blending, and optimizing mechanical recycling strategies.

## Figures and Tables

**Table 1:** Summary of polymer properties used in this study.

Sample Type	Soluble Fraction (%)	Melt Index (g/10 min)	Mw (g/mol)	MWD (M_w_/M_n_)	CH_3_/1000TC
C4-LLDPE	10	1.0	131,300	4.8	25
Homo-PP(1)	4	1.5	425,500	6.6	338
Homo-PP(2)	2	2.3	408,000	8.8	339
Random-PP(1)	6.4	1.9	366,400	4.9	320
Random-PP(2)	7.4	11	250,700	5.9	321

## Data Availability

Data will be made available on request.

## Supplementary Materials

The following supporting information can be downloaded at: https://www.mdpi.com/article/10.3390/polym17182543/s1.
