# Supplementary material for: Polymer Blend Spiking Method for Quantifying Polypropylene Variants in 100% Polypropylene Blends
Source: Polymers (Basel). 2025 Sep 20;17(18):2543. doi: 10.3390/polym17182543 (PMC12473938; doi:10.3390/polym17182543)
Supplement: Supplementary file 1 [file polymers-17-02543-s001.zip › polymers-3881336-supplementary.pdf]

Supporting Information

Polymer Blend Spiking Method for Quantifying Polypropylene Variants in 100% Polypropylene Blends

*Meysam Hashemnejad *, and Ami Doshi*

LyondellBasell, Cincinnati Technology Center, 11530 Northlake Drive, Cincinnati, Ohio 45249
meysam.hashemnejad@lyondellbasell.com

As shown in Fig. S1, the DSC results for the Random-PP and Homo-PP blends exhibit a gradual and continuous shift in both crystallization and melting behaviors as the composition transitions from 100% Random-PP to 100% Homo-PP. As expected, single peaks are observed in both the crystallization and melting thermograms, with corresponding enthalpy values decreasing slightly across the series from Homo-PP to Random-PP. This smooth progression indicates that Random-PP and Homo-PP are not only miscible but also co-crystallize and co-melt within the blend matrix. Consequently, the thermal events do not resolve into distinct peaks for each component, making it impractical to quantitatively deconvolute the individual contributions of Random-PP and Homo-PP from the DSC thermograms. This behavior highlights a high degree of compatibility and interaction between the two polypropylene types, which is beneficial for tuning material properties but limits straightforward phase quantification using DSC alone.

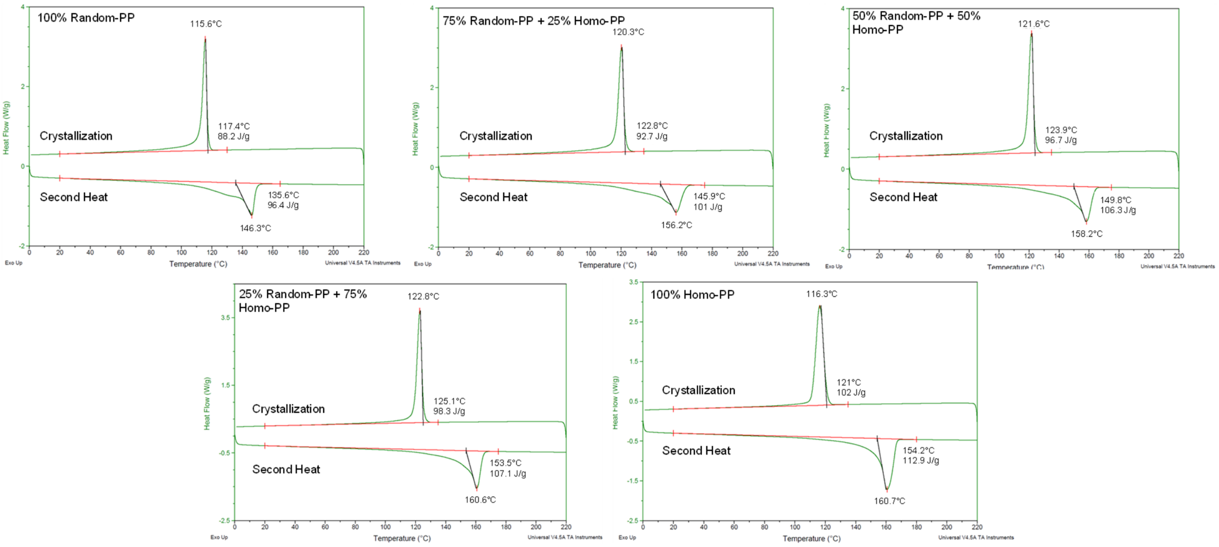

Figure S1. Differential Scanning Calorimetry (DSC) thermograms of Random-PP and Homo-PP blends showing crystallization and melting behavior across compositions ranging from 100% Random-PP to 100% Homo-PP. The presence of single, gradually shifting peaks in both crystallization and melting regions indicates co-crystallization and co-melting of the two polypropylene types, making phase quantification impractical.
